# The metabolic waste ammonium regulates mTORC2 and mTORC1 signaling

**DOI:** 10.1038/srep44602

**Published:** 2017-03-17

**Authors:** Ahmad Merhi, Paul Delrée, Anna Maria Marini

**Affiliations:** 1 Biology of Membrane Transport, IBMM, Université Libre de Bruxelles, Rue des Professeurs Jeener et Brachet 12, 6041 Gosselies, Belgium; 2 Institute of Pathology and Genetics, Avenue Georges Lemaître 25, 6041 Gosselies, Belgium; 3 Tumour Bank, Institute of Pathology and Genetics, Avenue Georges Lemaître 25, 6041 Gosselies, Belgium

## Abstract

Two structurally and functionally distinct mammalian TOR complexes control cell growth and metabolism in physiological and pathological contexts including cancer. Upregulated glutaminolysis is part of the metabolic reprogramming occurring in cancer, providing fuels for growth but also liberating ammonium, a potent neurotoxic waste product. Here, we identify ammonium as a novel dose-dependent signal mediating rapid mTORC2 activation and further regulating mTORC1. We show that ammonium induces rapid RICTOR-dependent phosphorylation of AKT-S473, a process requiring the PI3K pathway and further involving the Src-family kinase YES1, the FAK kinase and the ITGβ1 integrin. Release of calcium from the endoplasmic reticulum store triggers rapid mTORC2 activation, similar to ammonium-induced activation, the latter being conversely prevented by calcium chelation.Moreover, in analogy to growth factors, ammonium triggers the AKT-dependent phosphoinhibition of the TSC complex and of PRAS40, two negative regulators of mTORC1. Consistent with mTORC1 stimulation, ammonium induces the inhibitory phosphorylation of 4EBP1, a negative regulator of protein biogenesis. Ammonium however dually impacts on the phosphorylation of p70S6K1 triggering a transient AKT-independent decrease in the phosphorylation of this second mTORC1 readout. Finally, we reveal ammonium as a dose-dependent stimulator of proliferation. This study underscores an mTORC2 and mTORC1 response to the so-called ammonium waste.

TOR (target of rapamycin) is a serine/threonine kinase conserved in all eukaryotes that integrates extra and intracellular signals with cell growth^1^. Mammalian TOR is part of two distinct complexes. mTORC1 responds to nutrients, energy levels and growth factors and stimulates translation and anabolic metabolism while inhibiting autophagy. In contrast little is known about mTORC2 regulation. The latter complex responds mainly to growth factors via PI3K signaling, phosphorylates members of the AGC kinases family, including AKT, all linked to cancer and diabetes^2^,^3^. Though ‘cancer’ represents a puzzling number of at least 100 diseases, a set of common characteristics indicate important shared metabolic variations^4^,^5^. In many cancers, genetic alterations lead to constitutive activation of mTOR signaling, impacting on tumor cell metabolism^1^,^6^. One aspect of this metabolic reprogramming concerns the enhanced rate of glutamine transport and the upregulated catabolism of this amino acid, two processes currently targeted by therapeutic strategies^4^,^7^,^8^. In the mitochondria, glutamine is first deaminated by glutaminase (GLS) to glutamate that can in turn be converted to α-ketoglutarate, feeding the TCA cycle^5^. Increased glutaminolysis has been linked to oncogenic levels of Myc through a coordinated transcriptional program enhancing GLS expression^9^. High rate glutaminolysis is also expected to liberate high levels of ammonium. NH_4_^+^ is in pH-dependent equilibrium with NH_3_ and at pH7 for instance the ionic molecule largely predominates (>98%) over the neutral form. Hereafter, the term ‘ammonium’ will refer to the sum of NH_4_^+^ and NH_3_ unless a molecular distinction is required and ‘ammonia’ will refer to NH_3_. Accordingly, the seminal work by Chance and collaborators reported elevated ammonium levels in the blood of tumor-bearing rats^10^,^11^. Ammonium concentrations of up to 5 mM were subsequently found in the interstitial fluids from human tumor xenografts^12^. Although the importance of glutamine as a tumor nutrient is recognized since the 1950’s, the potential side-consequences of ammonium produced by glutaminolysis in tumors have been poorly documented. Ammonia was proposed to behave as an autocrine and paracrine diffusible factor stimulating autophagy in tumor masses through an unknown pathway independent of mTORC1 inhibition, and offering in turn a potential survival way to cells lacking nutrients and/or oxygen^12^,^13^. A phosphoproteomic analysis supported that mTOR activity was not affected in response to 5 mM ammonium, but failed to identify the pathway responsible for ammonium-induced autophagy^14^. A negative impact of ammonium on mTORC1 activity was recently reported, with a sustained inhibition in the presence of 10 mM ammonium, leading to reconsider the involvement of mTORC1 in ammonium-induced autophagy^15^. In a non pathological situation, ammonium mainly emerges from the catabolism of proteins and the activity of the intestinal flora. It is maintained at low plasmatic level, below 50 μM, thanks to hepatic metabolism mediating its conversion principally into urea via the urea cycle and for a minor part into glutamine via the glutamine synthetase (GS) activity. The impairment of ammonium detoxification occurring in case of liver dysfunction can lead to hyperammoniaemia, subsequent development of hepatic encephalopathy and, in acute cases, to lethal cerebral paralysis^16^. Ammonium plays in parallel a key role in the regulation of the acid-base balance^17^. The daily acid overload must imperatively be eliminated to ensure blood pH homeostasis. Activation of renal production of ammonium from glutamine breakdown by GLS and subsequent urinary elimination of ammonium is crucial for pH control.

Ammonium is also a key nutrient on earth, serving as a principal nitrogen source for most microbes and plants^18^. In baker yeast for instance, ammonium can be used as sole nitrogen supply and constitutes with glutamine a preferred nitrogen source stimulating growth by activating TORC1^19^,^20^,^21^.

Here, we show that ammonium triggers rapid and sensitive mTORC2-dependent phosphorylation of AKT-S473 in cancer cells. We show that the latter mTORC2 activation occurs via the PI3K pathway and relies on YES1 and FAK kinases, on integrin ITGβ1 and on calcium stores mobilization. In addition, our data indicate that ammonium also leads to an AKT-dependent stimulation of mTORC1 signaling and to a dose-dependent stimulation of proliferation. Our results thus identify the so-called ammonium waste as a signal impacting on mTORC2 and mTORC1 activity.

## Results

### Ammonium induces dose and time dependent AKT S473 phosphorylation

**Figure 1:**
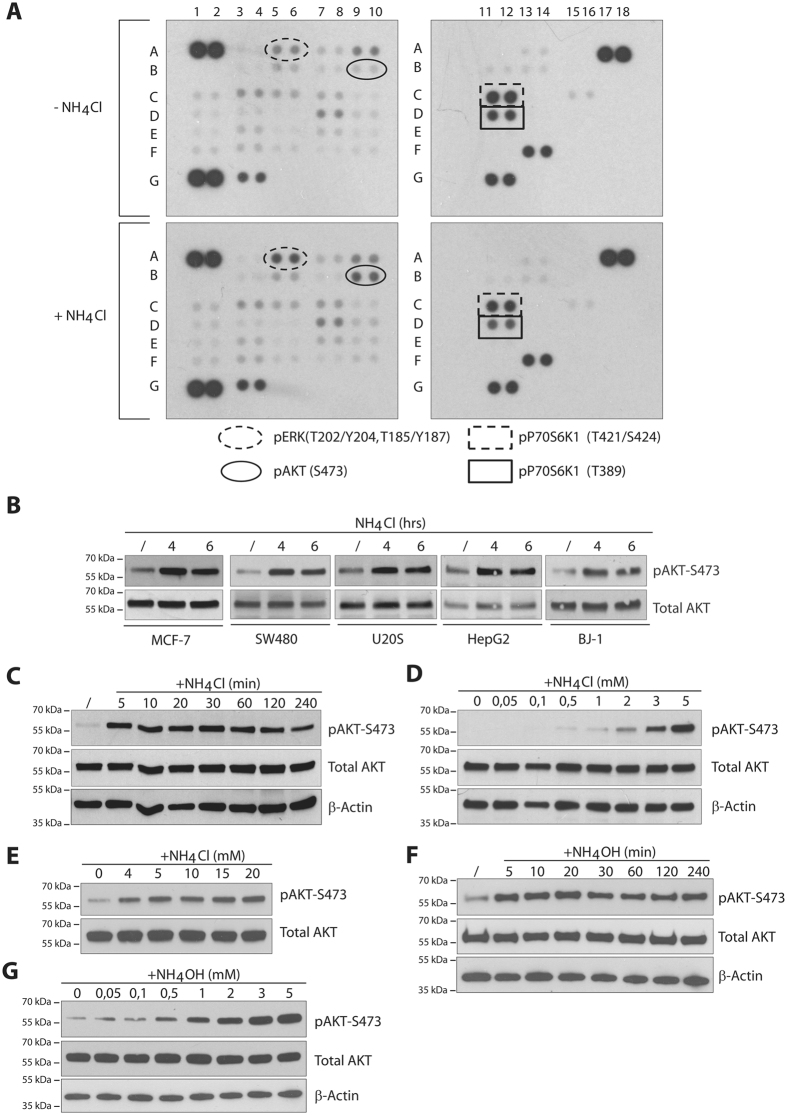
Ammonium induces dose and time dependent AKT phosphorylation. (**A**) A phospho-proteins kinase array was probed with lysates of MCF-7 cells treated or not with NH_4_Cl (5 mM) for 30 minutes. The dots corresponding to pAKT, pERK and p70S6K1 are indicated. (**B**) MCF-7, U2OS, HepG2, SW480 or BJ-1 cells were treated with NH_4_Cl (5 mM) for the indicate time. (**C**) MCF-7 cells were treated with NH_4_Cl (5 mM) for different time intervals. (**D** and **E**) MCF-7 cells were treated with the indicated NH_4_Cl concentrations for 30 minutes. (**F**) MCF-7 cells were treated with NH_4_OH (5 mM) for different time intervals. (**G**) MCF-7 cells were treated with the indicated NH_4_OH concentrations for 30 minutes. Lysates were immunoblotted with the indicated antibodies. Uncropped images of immunoblots are shown in Supplementary Figure S1.

To highlight potential signaling pathways responding to ammonium, we probed a phospho-kinase array with lysates of MCF-7 breast cancer cells treated or not with 5 mM NH_4_Cl for 30 minutes (Fig. 1A). We used a 5 mM NH_4_Cl concentration in reference to the highest ammonium concentration measured in tumor xenografts, a concentration that is reported to induce the autophagic process^12^. Of note, the latter concentration is far below those often used *in vitro* to inhibit the lysosomal function^22^,^23^,^24^. In these conditions, we detected a prominent increase in the phosphorylation of AKT-S473. In addition, we also noted an increased ERK1/2 phosphorylation (Fig. 1A). The latter ERK phosphorylation is consistent with previous data obtained in a phosphoproteomic analysis^14^. We decided to focus on the analysis of the uncharacterized AKT-S473 phosphorylation.

We confirmed by western blot that 5 mM NH_4_Cl induces the S473 phosphorylation of the AKT/PKB readout in MCF-7 cells. We similarly observed AKT phosphorylation in U2OS osteosarcoma, HepG2 hepatoma and SW480 colon adenocarcinoma cells (Fig. 1B). Moreover, AKT-S473 phosphorylation was also observed in the BJ-1 fibroblast cell line (Fig. 1B). These data indicate that 5 mM NH_4_Cl mediates common signaling pathways activation in cancer and fibroblast cell lines. NH_4_Cl-induced AKT phosphorylation occurred rapidly, with a maximal immunodetection at the earliest time-point of 5 min treatment and tended to decrease at 240 min (Fig. 1C). AKT phosphorylation was immunodetected with NH_4_Cl concentrations far lower than 5 mM, already visible at 0.5 mM, suggesting the stimulation of a sensitive pathway rapidly responding to low NH_4_Cl concentrations, far below those inhibiting the lysosomal function (Fig. 1D). AKT phosphorylation was observed for NH_4_Cl concentrations higher than 5 mM as well (Fig. 1E). Eng and co-workers showed that ammonium supplied as either NH_4_Cl or NH_4_OH was efficient in stimulating autophagy, while other molecules as NaOH, CO_2_, NO, H_2_O_2_ and lactic acid were inefficient^12^. Here, use of NH_4_OH, instead of NH_4_Cl, mediated a similar AKT-S473 phosphorylation both in terms of dose and time-dependencies (Fig. 1F and G). These data indicate that ammonium supplied as either NH_4_Cl or NH_4_OH leads to the induction of AKT-S473 phosphorylation in a time and dose dependent manner.

### Ammonium-induced AKT phosphorylation is dependent on mTORC2

**Figure 2:**
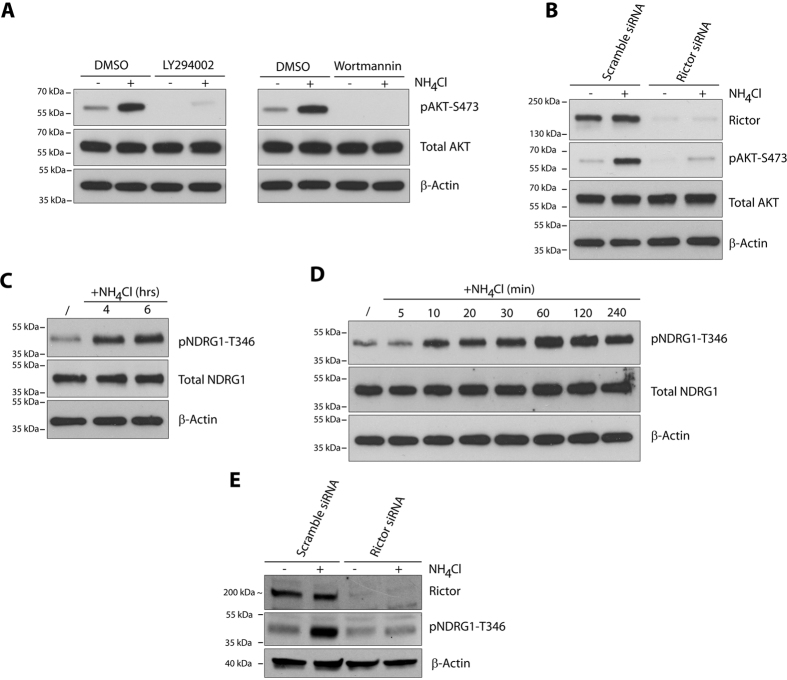
Ammonium-induced AKT phosphorylation is mTORC2-dependent. (**A**) MCF-7 cells were pretreated with DMSO, 50 μM LY29004 or 1 μM Wortmannin for 2 hours prior to NH_4_Cl (5 mM) addition for 30 minutes. (**B**) MCF-7 cells were transfected with scramble or RICTOR siRNA for 72 hours prior to NH_4_Cl (5 mM) addition for 30 minutes. (**C**) MCF-7 cells were treated with NH_4_Cl (5 mM) for 4 or 6 hours. (**D**) MCF-7 cells were treated with NH_4_Cl (5 mM) for the indicated time intervals. (**E**) MCF-7 cells were transfected with scramble or RICTOR siRNA for 72 hours prior to NH_4_Cl (5 mM) addition for 30 minutes. Lysates were immunoblotted with the indicated antibodies. Uncropped images of immunoblots are shown in Supplementary Figure S1.

We next dissected the upstream pathway leading to the rapid induction of AKT phosphorylation occurring upon NH_4_Cl supplementation. PI3K is largely described to transduce extracellular signals to activate AKT kinase^25^. Pretreatment of MCF-7 cells with LY294002 or Wortmannin to inhibit PI3K, impaired basal AKT phosphorylation level and prevented its induction by NH_4_Cl addition (Fig. 2A). The mTORC2, composed of mTOR, RICTOR, SIN1, PROTOR and DEPTOR, has been shown to promote cell proliferation and survival by phosphorylation and activation of the AGC kinase family members AKT and SGK^26^. In contrast to mTORC1, little is known about the signals regulating mTORC2. The phosphorylation of AKT at Ser-473 is so far the best characterized readout for mTORC2 activity^26^. We show that upon siRNA-mediated knockdown of RICTOR expression, the basal and the NH_4_Cl-induced AKT-S473 phosphorylation were strongly reduced (Fig. 2B). Moreover, NH_4_Cl was found to induce T346 phosphorylation of NDRG1, a specific target of SGK1, another reliable readout of mTORC2 signaling^26^, and the latter phosphorylation was dependent on mTORC2 (Fig. 2C,D and E). These data together support that ammonium stimulates PI3K/mTORC2/AKT signaling.

### Ammonium-induced mTORC2 activation is dependent on YES1 and FAK kinases and ITGβ1 integrin

**Figure 3:**
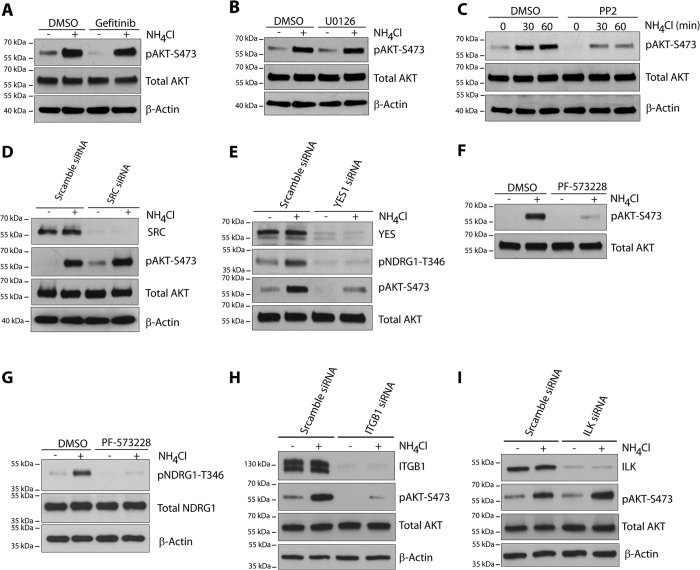
Ammonium-induced mTORC2 activation is dependent on YES1, FAK kinases and integrin β1. (**A**,**B**) MCF-7 cells were pretreated with DMSO, 10 μM Gefitinib, or 10 μM U0126 for 2 hours prior to NH_4_Cl (5 mM) addition for 30 minutes. (**C**) MCF-7 cells were pretreated with DMSO or 10 μM PP2 for 2 hours prior to NH_4_Cl (5 mM) addition for 30 or 60 minutes. (**D**,**E**) MCF-7 cells were transfected with scramble, YES1 or SRC siRNA for 72 hours prior to NH_4_Cl (5 mM) addition for 30 minutes. (**F**,**G**) MCF-7 cells were pretreated with DMSO or 10 μM PF573228 for 2 hours prior to NH_4_Cl (5 mM) addition for 30 minutes. (**H**,**I**) MCF-7 cells were transfected with scramble, integrin β1 (ITGB1) or ILK siRNA for 72 hours prior to NH_4_Cl (5 mM) addition for 30 minutes. Lysates were immunoblotted with the indicated antibodies. Uncropped images of immunoblots are shown in Supplementary Figure S1.

We next addressed how ammonium activates mTORC2. It is reported that epidermal growth factor receptor (EGFR) stimulates mTORC2 kinase activity and signaling^27^. The use of pharmacological inhibition of EGFR or the downstream MEK activity with Gefitinib and U0126 respectively indicated that NH_4_Cl–induced AKT-S473 phosphorylation occurred independently of EGFR or MEK kinases (Fig. 3A and B). In contrast, inhibiting SFK kinases by pretreatment of cells with PP2 reduced basal and NH_4_Cl-induced AKT-S473 phosphorylation (Fig. 3C), revealing a SFK requirement for NH_4_Cl-induced mTORC2 activation. Specific siRNA-mediated knockdown of SRC kinase did not impair NH_4_Cl-induced AKT-S473 phosphorylation (Fig. 3D). In contrast, knockdown of YES1 kinase decreased basal and both NH_4_Cl-induced AKT-S473 and NDRG1-T346 phosphorylation (Fig. 3E). Moreover, pharmacological inhibition with PF573228 of the focal adhesion kinase (FAK), reported to interact with mTORC2 but not mTORC1^28^, reduced NH_4_Cl -induced AKT-S473 and NDRG1-T346 phosphorylation (Fig. 3F and G). These results together indicate that YES1 and FAK kinases are involved in the NH_4_Cl-induced AKT phosphorylation. We also addressed whether integrins, known regulators of FAK signaling, could be involved in the NH_4_Cl-induced AKT-S473 phosphorylation^29^. We found that siRNA-mediated knockdown of integrin ITGβ1, but not of ILK, decreased the basal and the NH_4_Cl-induced AKT-S473 phosphorylation (Fig. 3H and I). Altogether our data indicate that ammonium-induced activation of mTORC2 involves ITGβ1, FAK, YES1, and PI3K signaling.

### Ammonium-induced mTORC2 activation is modulated by calcium

**Figure 4:**
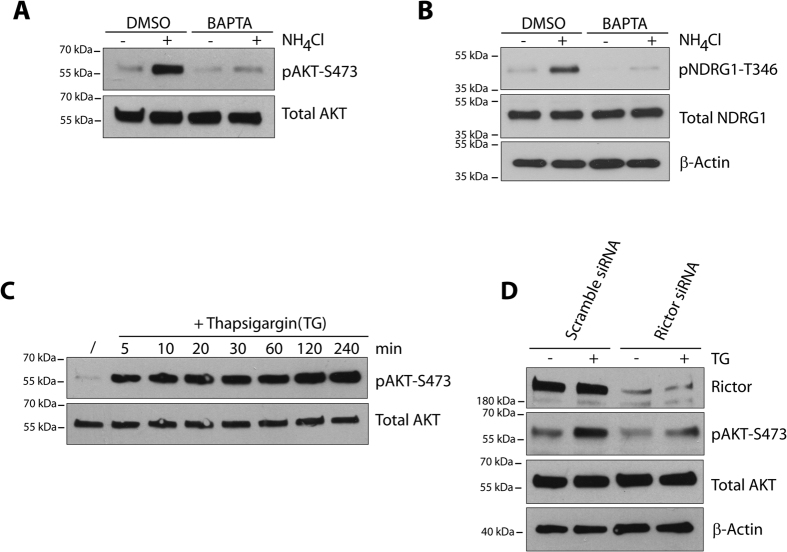
Ammonium-induced mTORC2 activation is modulated by calcium. (**A**,**B**) MCF-7 cells were pretreated with DMSO or 50 μM BAPTA for 1 hour prior to NH_4_Cl (5 mM) addition for 30 minutes. (**C**) MCF-7 cells were treated with Thapsigargin (TG) for the indicated times. (**D**) MCF-7 cells were transfected with scramble or RICTOR siRNA for 72 hours prior to TG addition for 30 minutes. Lysates were immunoblotted with the indicated antibodies. Uncropped images of immunoblots are shown in Supplementary Figure S1.

Our data reveal that ammonium supplied as NH_4_Cl or NH_4_OH can trigger a rapid and relatively sensitive phosphorylation of AKT-S473. This raises the question of what is the molecular entity that is initially sensed as activator. Is NH_4_^+^, or NH_3,_ directly sensed, or is it a derivative or a consequence of NH_4_^+^/NH_3_ entry in the cells? NH_4_Cl treatment has been shown to induce a rapid transient increase in intracellular Ca^2+^ in cultured astrocytes^30^. We thus assessed the potential role of calcium in NH_4_Cl-induced mTORC2 activation. Pretreatment of cells with the cell-permeable calcium chelator BAPTA-AM decreased basal and NH_4_Cl-induced AKT-S473 and NDRG1-T346 phosphorylation (Fig. 4A and B). In addition, treatment of cells with thapsigargin (TG), an inhibitor of endoplasmic reticulum Ca^2+^ -ATPase known to trigger an increase in cytoplasmic calcium concentration^31^, induced a rapid increase in AKT-S473 phosphorylation (Fig. 4C). The latter TG-induced phosphorylation of AKT was dependent on mTORC2 activity (Fig. 4D). These results together support that ammonium-induced mTORC2-dependent AKT phosphorylation is modulated by the mobilization of intracellular calcium stores.

### Ammonium induces AKT-dependent activation of mTORC1 signaling

**Figure 5:**
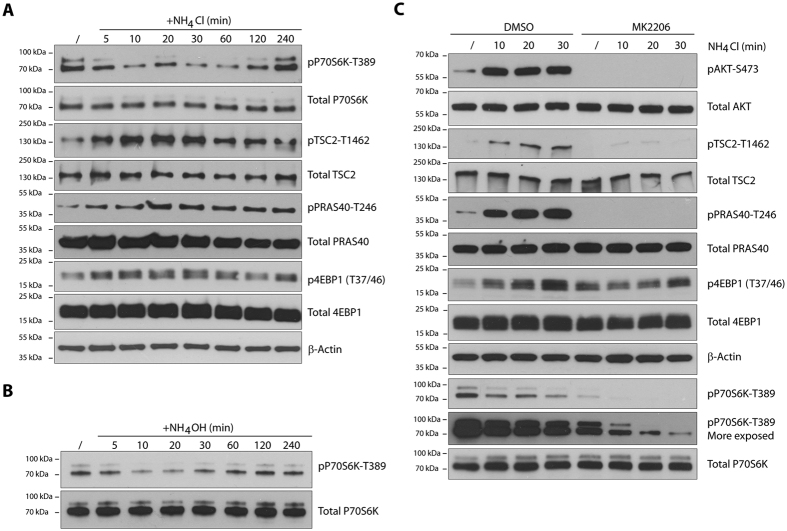
Ammonium induced mTORC1 kinase activation in AKT dependent manner. (**A**) MCF-7 cells were treated with NH_4_Cl (5 mM) for different time intervals. (**B**) MCF-7 cells were treated with NH_4_OH (5 mM) for different time intervals. (**C**) MCF-7 cells were pretreated with DMSO or 1 μM MK2206 for 10 minutes prior to NH_4_Cl (5 mM) addition for different time intervals. Lysates were immunoblotted with the indicated antibodies. Uncropped images of immunoblots are shown in Supplementary Figure S1.

The impact of ammonium on the activity of mTORC1, whether neutral or inhibitory, remains unclear^12^,^13^,^14^,^15^. In line with a potential mTORC1 inhibition by ammonium, the kinase array (Fig. 1A) also revealed a decreased phosphorylation of p70S6K-T389, an mTORC1 readout, after 30 minutes treatment with 5 mM NH_4_Cl. Western blot analysis of the time-course impact of NH_4_Cl on pP70S6K-T389 confirmed the slight dephosphorylation (Fig. 5A). This occurred rapidly after NH_4_Cl addition, being detectable at the earliest time point of 5 minutes. However, the weak dephosphorylation of p70S6K-T389 appeared transient, phosphorylation being restored at time 240 min, suggesting that the rapid detected inhibition of mTORC1 was quickly compensated by a reactivation. Moreover, 5 mM NH_4_OH, used in place of NH_4_Cl, provoked a similar transient dephosphorylation of p70S6K-T389 (Fig. 5B). Although, we do not exclude an osmotic effect, these data suggest that ammonium induces a rapid but transient inhibition of mTORC1 activity.

We next wished to address the mechanism of mTORC1 activity recovery following the rapid inhibition. Activation of mTORC1 by growth factors is predominantly mediated through the PI3K-AKT pathway^2^,^32^. AKT activation has been shown to promote mTORC1 by inhibiting the TSC complex, a negative regulator of mTORC1^32^. Furthermore, AKT-mediated phosphorylation of another negative regulator of mTORC1, PRAS40, prevents its inhibitory role^32^,^33^. Given that ammonium promotes AKT signaling, we further investigated the effect of NH_4_Cl 5 mM on mTORC1 activation. We found that NH_4_Cl induced the rapid phosphorylation of TSC2-T1462, peaking during the first 30 minutes (Fig. 5A). It also induced rapid phosphorylation of PRAS40-T246 suggesting consequent rapid mTORC1 activation (Fig. 5A). The apparent mTORC1 activation however contrasts with the decreased phosphorylation of p70S6K-T389 that is rapidly observed after NH_4_Cl addition. We therefore tested another readout of mTORC1 activity, the phosphorylation of 4EBP1 (T37/46). Phosphorylation of 4EBP1 (T37/46) inhibits this negative regulator of translation thereby allowing protein biogenesis^34^. In this case, NH_4_Cl addition induced the rapid inhibitory phosphorylation of 4EBP1 (T37/46), consistent with mTORC1 activation (Fig. 5A). These data indicate that an additional regulatory process occurs upon NH_4_Cl addition that transiently counteracts the mTORC1-mediated stimulation of p70S6K-T389 phosphorylation.

Treatment with MK2206, a highly selective and potent inhibitor of AKT^35^, not only prevented the NH_4_Cl-induced phosphorylation of AKT, but also reduced the phosphorylation of TSC2, PRAS40 and 4EBP1, consistent with NH_4_Cl-induced activation of mTORC1 being AKT-dependent (Fig. 5C). Moreover, MK2206 inhibition of AKT did not impair the rapid NH_4_Cl-induced dephosphorylation of p70S6K-T389 (Fig. 5C). This data indicates that the additional regulatory process stimulated by ammonium, possibly corresponding to a phosphatase activation, is AKT-independent.

Collectively, these data are consistent with ammonium promoting AKT-dependent mTORC1 activation in addition to mTORC2 stimulation.

### Ammonium stimulates cell proliferation in a dose-dependent manner

**Figure 6:**
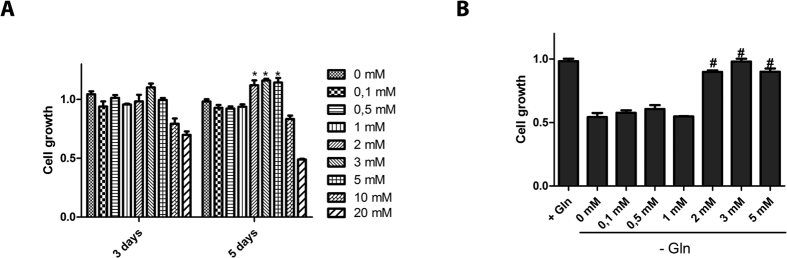
Ammonium stimulates cell proliferation in a dose-dependent manner. (**A**) MCF-7 cells were treated with the indicated NH_4_Cl concentrations for 3 or 5 days and proliferation was determined by crystal violet staining. (**B**) Cells were either in culture medium with 2 mM glutamine or glutamine-free medium and supplemented with the indicated concentration of NH_4_Cl. Cell growth was determined by crystal violet staining.

Finally, we evaluated the dose-dependent impact of NH_4_Cl on the proliferation of MCF-7 cells. Addition of 10 and 20 mM NH_4_Cl resulted in a significant cell growth inhibition after 3 and 5 days while NH_4_Cl concentrations below 5 mM had no negative impact on proliferation (Fig. 6A). In contrast, addition of 2, 3 or 5 mM NH_4_Cl resulted in slight but significant increase in cell proliferation. Moreover, the latter NH_4_Cl doses were able to rescue the growth defect of MCF-7 cells cultured in glutamine-free medium (Fig. 6B).

These data indicate that the so-called ammonium waste can act as a dose-dependent stimulator of proliferation.

## Discussion

**Figure 7:**
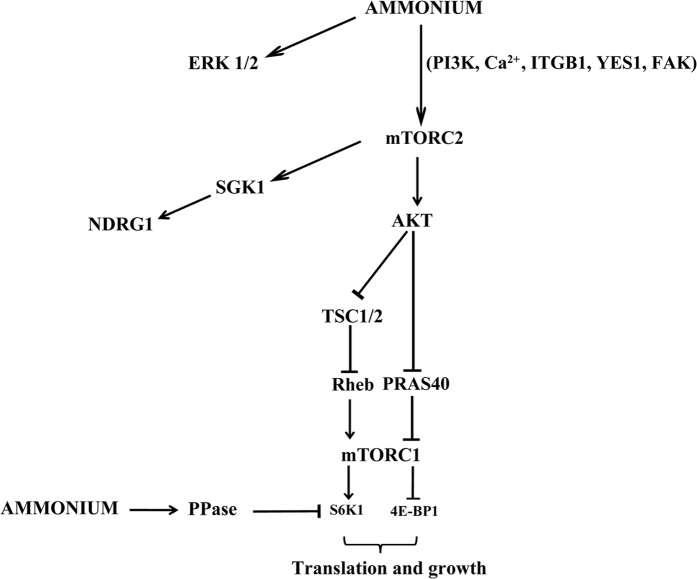
Model of the multifaceted response to ammonium. Ammonium triggers mTORC2 activation which phosphorylates and activates AKT in integrinβ1, YES1 (Src family kinase), and FAK (Focal adhesion kinase) dependent manner. In addition, activated AKT phosphorylates and inhibits TSC2 in the TSC complex and PRAS40, relieving the TSC-mediated repression of Rheb and Raptor-PRAS40 interaction respectively, thereby increasing mTORC1 signaling. Active mTORC1 phosphorylates and inhibits 4E-BP1. Once phosphorylated by mTORC1, 4E-BP1 releases eIF4E and stimulates translation initiation. We further propose that ammonium activates, yet to identify, phosphatases mediating the dephosphorylation of p70S6K1. In parallel to mTORC2 and mTORC1 stimulation, ammonium also promotes ERK signaling.

This study identifies ammonium as promoting both mTORC2 and mTORC1 signaling and brings insights into the molecular mechanism of the ammonium-mediated regulation (Fig. 7). In contrast to the many cues activating mTORC1, mTORC2 is mainly documented for being activated by growth factors^2^,^26^. However, recent advances, including observations made in the yeast model, indicate that mTORC2 can be activated by additional signals as well. Membrane tension has been reported to promote yeast TORC2 and mTORC2 activation, the latter enabling for instance a control of cell polarity and mobility^3^,^36^,^37^,^38^,^39^. On the other hand, methylglyoxal, an intermediate of glycolysis, was also recently reported to activate yeast TORC2 and mTORC2^40^. Our study now indicates that mTORC2 is further linked to the metabolic status by responding to ammonium, a byproduct of glutaminolysis. How mTORC2 activation is regulated is also not fully understood^3^. Growth factors were shown to promote, via PI3K signaling, association of mTORC2 to the ribosome and its activation^41^. Like for the activation of mTORC2 mediated by growth factors, the ammonium-induced activation turned to be dependent on PI3K, whereas it was independent of the growth factor receptor EGFR and MEK kinase. Of note, our study identifies YES1, a kinase of the Src family, as involved in the ammonium-induced phosphorylation of both AKT-S473 and NDRG1-T346 mTORC2 readouts. SFK kinases have been shown to participate to cancer cell proliferation and survival, YES1 being currently considered as a potential therapeutic target in a number of cancers^42^. We additionally found that the focal adhesion kinase FAK, a partner of mTOR2 but not of mTOR1, implicated in survival, migration and invasion^43^, is also involved in the ammonium-induced phosphorylation of mTORC2 readouts. Interestingly, YES1 and FAK interaction has been reported in melanoma, suggesting that both proteins could work in a concerted manner^44^. The transmission of the ammonium signal leading to AKT-S473 phosphorylation also implicated the β1-integrin, known to activate FAK signaling both in normal and cancer cells^45^,^46^.

Our data are thus consistent with ammonium-induced activation of AKT relying on β1-integrin, FAK, YES1, PI3K and mTORC2 (Fig. 7). Interestingly, β1-integrin/FAK/PI3K/AKT/mTOR signaling was recently implicated in the metabolic reprogramming favoring glycolysis in human mammary epithelial cells overexpressing Twist, a key transcription factor for epithelial-mesenchymal transition and thus invasion and metastasis^47^. Whether ammonium-mediated signalling to mTORC2 can lead to glycolysis activation remains to be evaluated. It is interesting to note that glutamine metabolism, as well as exogenous addition of ammonium, under normoxic conditions were reported to lead to the stabilization of the HIF1α transcription factor known to favor the transcription of several genes involved in glycolysis under hypoxic conditions^48^,^49^.

Our data also indicate that ammonium promotes mTORC1 signaling. Different pathways convey the signals enabling mTORC1 activation^1^,^32^. Conserved Rag GTPases for instance control the amino acids-dependent TORC1 signaling^50^. On the other hand, growth factors activate mTORC1 indirectly by suppressing the inhibitory effect of negative regulators, the TSC complex and PRAS40. Activated AKT phosphorylates and inhibits TSC2 in the TSC complex and PRAS40, relieving the TSC-mediated repression of Rheb and Raptor-PRAS40 interaction respectively, thereby increasing mTORC1 signaling^32^. We found that ammonium increased AKT-dependent phosphorylation of both TSC2 and PRAS40, consistent with mTORC1 activation. Indeed, ammonium induced the phosphorylation of the mTORC1 downstream target 4EBP1 suggesting that ammonium promoted protein biogenesis. Intriguingly, and as recently reported^15^, we observed an inhibitory impact of ammonium on the phosphorylation level of a second mTORC1 substrate, the p70S6K1 kinase. However, the weak dephosphorylation of p70S6K1 appeared to be transient and is AKT-independent. This observation could be explained by ammonium promoting, in addition to mTORC1, the activation of phosphatases favoring p70S6K1 dephosphorylation as a rapid response to ammonium. For instance, in the Drosophila model, S6K is dephosphorylated by PP2A phosphatase and this is implicated in the response to the nutritional conditions^51^. The PHLPP phosphatase is also reported to dephosphorylate p70S6K1^52^. Osmotic stress was recently shown to mediate a transient inactivation of mTORC1^53^. However, in this case the response was observed with NaCl concentration above 25 mM, thus well above the osmotic perturbation caused by 5 mM NH_4_Cl or NH_4_OH used in our experimental set-up. In addition, the osmotic stress affected both p70S6K1 and 4EPB1 mTORC1 substrates and it also transiently reduced the AKT-S473 and the ERK1/2 phosphorylation which contrasts with our observations with NH_4_Cl treatment. However, the decrease in p70S6K1 phosphorylation was found to be both TSC2/Rheb dependent and independent, and Calyculin-A-sensitive phosphatases were proposed to directly dephosphorylate p70S6K1 in response to the osmotic stress via an unknown pathway. Further investigations are needed to determine the mechanism of p70S6K1 dephosphorylation after ammonium addition.

We also found that calcium mobilization is required for ammonium-induction of mTORC2 signaling. Treatment with thapsigargin to enable the release of calcium from ER was sufficient to observe rapid mTORC2-dependent phosphorylation of AKT-S473, as similarly observed with ammonium. Growth hormones were shown to trigger the localization of mTORC2 at the mitochondrial-associated ER membrane where it controls aspects of the mitochondrial physiology, including metabolism and calcium transport^54^. Moreover, calcium was recently suggested to modulate both mTORC1 and mTORC2 in response to growth factors^55^. Our data in turn indicate that ammonium-induced activation of mTORC2 can be modulated by calcium. It is conceivable that ammonium affects the calcium stores via a pH-mediated effect. Ammonium is indeed reported to induce calcium release from the lysosome^30^. Ammonium is a widely used molecule to alter lysosomal function, by inducing a pH raise inhibiting proteases activity^56^. Ammonium has even been reported to inhibit rapamycin-induced autophagy in hepatocarcinoma HepG2 cells^57^. However, in these cases, ammonium concentrations used are above 10 mM, thus far above the levels found in tumor xenografts^10^,^11^,^12^. Ammonia was proposed to act as a diffusible signal triggering autophagy in distinct tumor regions^12^. The mechanism enabling ammonium-induced autophagy remains unclear^12^,^14^,^15^. As confirmed by our observations, ammonium was found to stimulate ERK1/2 phosphorylation^14^ (Fig. 7). However, the ERK pathway was not highlighted as required to initiate ammonium-induced autophagy^14^. It is likely that in growing tumor where cells are engaged in active glutaminolysis, the progressive production and accumulation of ammonium will reach levels that stimulate autophagy and also activate pro-survival signaling pathways leading to stimulate proliferation. This could occur in an autocrine and paracrine fashion. It was recently reported that the glutamine required for the growth of glioblastoma tumors is provided by the glutamine synthetase activity of GS-positive glioma cells in the tumor or by normal astrocytes^58^,^59^. GS enabled glutamine synthesis from ammonium and glutamate, fueling nucleotide biosynthesis and growth of glioblastoma. Supplementation of both GS substrates rescued the growth of glutamine-deprived cells. The shuttle of ammonium between cells with complementary metabolic factories, or from the circulation to the glutamine-synthesizing cells, could play a key role in tumor growth. Interestingly, our data show that while high ammonium concentrations inhibit the proliferation of MCF-7 breast cancer cells, doses in the 2–5 mM range can stimulate growth both in the presence or absence of glutamine supplementation. Ammonium, likely used to feed glutamine synthesis in glutamine-starved cells, appears to further stimulate proliferation of glutamine-supplemented cells. High ammonium concentrations probably affect several signaling pathways, and inhibit autophagy in this case, overall ending to an inhibition of proliferation, at least *in vitro*.

While ammonium is largely documented as a toxic waste product in animals, it is a major nitrogen source in micro-organisms and plants^16^. It is a preferred nitrogen source in yeast activating TORC1 and sustaining fast growth and proliferation^19^,^20^,^21^. Ammonium participates to the general control of the expression of transporters and enzymes involved in the utilization of non-preferred nitrogen sources^60^ and also triggers the inhibition of nutrient transport activity^61^,^62^,^63^. In addition, ammonium is a signal limiting yeast colonies overgrowth^64^, while ammonium limitation enables filamentous invasive growth^65^. In contrast, ammonium excess is also known to limit plant growth and to be toxic for yeast cells^66^. Hence, like further evidenced in this study, ammonium appears to have several dose-dependent effects in many domains of life.

Of note, although ammonium is widely assumed to be mainly transported across cell membranes by passive diffusion of ammonia, several transport proteins can mediate either specific or non-specific ammonium transport. For instance, a widely conserved protein family, comprising the human Rhesus factors, act as specific ammonium transport proteins^67^,^68^,^69^. We have recently shown that one of the Rhesus factors genes is upregulated in certain cancer cells^70^. Whether targeting ammonium transport proteins could be used as therapeutic strategy remains to be evaluated.

As part of their metabolic reprogramming, cancer cells liberate waste products, like lactate and protons derived from the upregulated glycolysis in the Warburg effect for instance^71^,^72^. Overall, our data reveal how another waste product of cancer cells, ammonium derived from upregulated glutaminolysis, could turn advantageous for proliferation by triggering key signaling pathways promoting growth.

## Experimental Procedures

### Cell culture

The human osteosarcoma U2OS, human hepatocellular carcinoma (HepG2), human colon adenocarcinoma (SW480), human fibroblast (BJ-1) were cultured in advanced DMEM medium (Invitrogen). The human breast cancer cell line MCF-7 was cultured in DMEM:F12 medium (Invitrogen). The media were supplemented with 10% fetal bovine serum (Biowest), 2 mM L-glutamine, 50 units/ml penicillin, and 50 μg/ml streptomycin. Cells were maintained in an incubator with humidified air (5% CO_2_) at 37 °C.

In all the experiments, cells were plated 1 day before treatment. The next day, the medium was replaced with a fresh one and cells were treated with either NH_4_Cl or NH_4_OH for the indicated time interval and with the indicated concentration.

### Reagents and antibodies

LY294002 (Cell Signaling, #9901) and Bapta-AM (Selleckchem, #S7534) were used at a 50 μM concentration. PP2 (Cayman Chemical, #13198–1), U0126 (Cell signaling, #9903), PF-573228 (Sigma, #PZ0117), Gefitinib (Invivogen, #tlrl-gef) were used at 10 μM concentration. Thapsigargin (Cayman Chemical, #10522) was used at 5 μM concentration. Wotrmannin (Cell Signaling, #9951) and MK-2206 2HCl (Selleckchem, #S1078) were used at 1 μM concentration.

Crystal violet was purchased from Sigma (#3886).

Antibodies for AKT (#4691), p-AKT-S473 (#4060), p-NDRG1-T346 (#5482), NDRG1 (#9485), p-P70S6K-T389 (#9234), P70S6K (#2708), RICTOR (#2114), p-PRAS40-T246 (#2997), PRAS40 (#2691), TSC2 (#4308), p-TSC2-T1462 (#3617), 4EBP1 (#9644), p-4EBP1-T37/46 (#2855), ITGB1 (#9699), ILK (#3856), SRC (#2123), YES (#3201), were from Cell Signaling. Antibody against β-Actin (#A3854) was from Sigma.

### Phospho-Kinase array

The human phospho-kinase array kit (ARY003) was obtained from R&D. NH_4_Cl 5 mM was added for 30 minutes and the levels of phopsho-proteins in cell lysates were analyzed according to the manufacturer’s instructions.

### Western Blot

Total proteins were extracted using RIPA (25 mM Tris-HCl pH 7.6, 150 mM NaCl, 1% NP-40, 1% sodium deoxycholate, 0.1% SDS) lysis buffer supplemented with cocktail of phosphatase and protease inhibitors (Roche). After centrifugation, proteins were quantified using Pierce Microplate BCA Protein Assay Kit – Reducing Agent Compatible assay (Thermo Scientific). Equal amounts (~15 μg) of proteins were then separated by Mini-PROTEAN TGX gels (Bio-Rad) and transferred to nitrocellulose membrane (Protran, VWR). Membranes were blocked with 5% milk and incubated overnight with the indicated antibodies. Primary antibodies were detected with horseradish-peroxidase-conjugated anti-rabbit or anti-mouse-IgG secondary antibodies (GE Healthcare) followed by measurement of chemoluminescence (Lumi-LightPLUS, Roche).

### RNA interference

Cells were revers transfected with pre-designed silencer select targeting RICTOR (Invitrogen, #s226000), YES1 (Invitrogen, #s14956), SRC (Invitrogen, #s13414), ITGB1 (Invitrogen, #s7574), ILK (Invitrogen, #s7404) or non-targeting control (Invitrogen, #4390843) with Lipofectamine siRNAMAX (Invitrogen) according to the manufacture’s protocol. Cells were incubated at 37 °C for 72 hours. NH_4_Cl 5 mM was then added or not for the indicated times and total protein extracts were immunoblotted with the indicated antibodies.

### Cell proliferation assay

For cell proliferation assays, 2 × 10^4^ cells were seeded in 24-well plates and cultured overnight. For glutamine-free conditions, cells grown with glutamine-containing medium were washed three times with pre-warmed glutamine-free medium and were then cultured in glutamine-free medium supplemented with NH_4_Cl at the indicated concentration for the indicated time. Cells were washed with PBS, fixed with 4% paraformaldehyde for 10 minutes and then stained with 0.1% crystal violet for 20 minutes at room temperature. Cells were washed 5 times with water and staining was extracted using 10% acetic acid. Absorbance was measured at 590 nm. All proliferation experiments were done in triplicates.

### Statistical analysis

Data are expressed as means ± S.E.M. of a triplicate. Statistical comparisons are assessed by Student’s *t*-tests using Graph Pad Prism version 5.00 software (Graph Pad Software). Differences are considered significant when the p value is below 0.05 (*p < 0.05, ^#^p < 0.001), n = 3.

## Additional Information

**How to cite this article:** Merhi, A. *et al*. The metabolic waste ammonium regulates mTORC2 and mTORC1 signaling. *Sci. Rep.* **7**, 44602; doi: 10.1038/srep44602 (2017).

**Publisher's note:** Springer Nature remains neutral with regard to jurisdictional claims in published maps and institutional affiliations.

## Supplementary Material

Supplementary Information

## References

[b1] Liko D. & Hall M. N. mTOR in health and in sickness. J. Mol. Med. (Berl). 93, 1061–73 (2015). 26391637 10.1007/s00109-015-1326-7

[b2] Shimobayashi M. & Hall M. N. Making new contacts: the mTOR network in metabolism and signalling crosstalk. Nat. Rev. Mol. Cell Biol. 15, 155–62 (2014). 24556838 10.1038/nrm3757

[b3] Gaubitz C., Prouteau M., Kusmider B. & Loewith R. TORC2 Structure and Function. Trends Biochem. Sci. 41, 532–45 (2016). 27161823 10.1016/j.tibs.2016.04.001

[b4] Vander Heiden M. G. Targeting cancer metabolism: a therapeutic window opens. Nat. Rev. Drug Discov. 10, 671–84 (2011). 21878982 10.1038/nrd3504

[b5] DeBerardinis R. J., Lum J. J., Hatzivassiliou G. & Thompson C. B. The biology of cancer: metabolic reprogramming fuels cell growth and proliferation. Cell Metab. 7, 11–20 (2008). 18177721 10.1016/j.cmet.2007.10.002

[b6] Ciriello G. . Emerging landscape of oncogenic signatures across human cancers. Nat. Genet. 45, 1127–1133 (2013). 24071851 10.1038/ng.2762 PMC4320046

[b7] Villar V. H., Merhi F., Djavaheri-Mergny M. & Durán R. V. Glutaminolysis and autophagy in cancer. Autophagy 11, 1198–208 (2015). 26054373 10.1080/15548627.2015.1053680 PMC4590661

[b8] Galluzzi L., Kepp O., Vander Heiden M. G. & Kroemer G. Metabolic targets for cancer therapy. Nat. Rev. Drug Discov. 12, 829–46 (2013). 24113830 10.1038/nrd4145

[b9] Dang C. V., Le A. & Gao P. MYC-induced cancer cell energy metabolism and therapeutic opportunities. Clin. Cancer Res. 15, 6479–83 (2009). 19861459 10.1158/1078-0432.CCR-09-0889 PMC2783410

[b10] Chance W. T., Cao L., Foley-Nelson T., Nelson J. L. & Fischer J. E. Possible role of ammonia in experimental cancer anorexia. Brain Res. 486, 316–24 (1989). 2731036 10.1016/0006-8993(89)90518-0

[b11] Chance W. T., Cao L., Nelson J. L., Foley-Nelson T. & Fischer J. E. Hyperammonemia in anorectic tumor-bearing rats. Life Sci. 43, 67–74 (1988). 3164438 10.1016/0024-3205(88)90238-x

[b12] Eng C. H., Yu K., Lucas J., White E. & Abraham R. T. Ammonia derived from glutaminolysis is a diffusible regulator of autophagy. Sci. Signal. 3, ra31 (2010). 10.1126/scisignal.2000911 20424262

[b13] Cheong H. & Lindsten T. Ammonia-induced autophagy is independent of ULK1/ULK2 kinases. **108** (2011). 10.1073/pnas.1107969108 PMC3131371 21690395

[b14] Harder L. M., Bunkenborg J. & Andersen J. S. A comparative phosphoproteomic study of the cellular response to ammonia and rapamycin Inducing autophagy. 1–17 (2014). 10.4161/auto.26863 PMC5396081 24300666

[b15] Li Z. . Ammonia Induces Autophagy through Dopamine Receptor D3 and MTOR. PLoS One 11, e0153526 (2016). 27077655 10.1371/journal.pone.0153526 PMC4831814

[b16] Auron A. & Brophy P. D. Hyperammonemia in review: pathophysiology, diagnosis, and treatment. Pediatr. Nephrol. 27, 207–22 (2012). 21431427 10.1007/s00467-011-1838-5

[b17] Weiner I. D. & Verlander J. W. Renal ammonia metabolism and transport. Compr. Physiol. 3, 201–20 (2013). 23720285 10.1002/cphy.c120010 PMC4319187

[b18] Von Wiren N. & Merrick M. In Mol. Mech. Control. transmembrane Transp. 95–120 (Topics in Current Genetics 9, 2004).

[b19] Godard P. . Effect of 21 different nitrogen sources on global gene expression in the yeast Saccharomyces cerevisiae. Mol. Cell. Biol. 27, 3065–86 (2007). 17308034 10.1128/MCB.01084-06 PMC1899933

[b20] Stracka D., Jozefczuk S., Rudroff F., Sauer U. & Hall M. N. Nitrogen source activates TOR (target of rapamycin) complex 1 via glutamine and independently of Gtr/Rag proteins. J. Biol. Chem. 289, 25010–20 (2014). 25063813 10.1074/jbc.M114.574335 PMC4155668

[b21] Cardenas M. E., Cutler N. S., Lorenz M. C., Di Como C. J. & Heitman J. The TOR signaling cascade regulates gene expression in response to nutrients. Genes Dev. 13, 3271–9 (1999). 10617575 10.1101/gad.13.24.3271 PMC317202

[b22] Maxfield F. R. Weak bases and ionophores rapidly and reversibly raise the pH of endocytic vesicles in cultured mouse fibroblasts. J. Cell Biol. 95, 676–81 (1982). 6183281 10.1083/jcb.95.2.676 PMC2112942

[b23] Seglen P. O. & Reith A. Ammonia inhibition of protein degradation in isolated rat hepatocytes. Quantitative ultrastructural alterations in the lysosomal system. Exp. Cell Res. 100, 276–80 (1976). 939253 10.1016/0014-4827(76)90148-8

[b24] Seglen P. O., Grinde B. & Solheim A. E. Inhibition of the lysosomal pathway of protein degradation in isolated rat hepatocytes by ammonia, methylamine, chloroquine and leupeptin. Eur. J. Biochem. 95, 215–25 (1979). 456353 10.1111/j.1432-1033.1979.tb12956.x

[b25] Bozulic L. & Hemmings B. A. PIKKing on PKB: regulation of PKB activity by phosphorylation. Curr. Opin. Cell Biol. 21, 256–61 (2009). 19303758 10.1016/j.ceb.2009.02.002

[b26] Oh W. J. & Jacinto E. mTOR complex 2 signaling and functions. Cell Cycle 10, 2305–16 (2011). 21670596 10.4161/cc.10.14.16586 PMC3322468

[b27] Tanaka K. . Oncogenic EGFR signaling activates an mTORC2-NF-κB pathway that promotes chemotherapy resistance. Cancer Discov. 1, 524–38 (2011). 22145100 10.1158/2159-8290.CD-11-0124 PMC3229221

[b28] Dey-Guha I. . A mechanism for asymmetric cell division resulting in proliferative asynchronicity. Mol. Cancer Res. 13, 223–30 (2015). 25582703 10.1158/1541-7786.MCR-14-0474 PMC4336804

[b29] Harburger D. S. & Calderwood D. A. Integrin signalling at a glance. J. Cell Sci. 122, 159–63 (2009). 19118207 10.1242/jcs.018093 PMC2714413

[b30] Rose C., Kresse W. & Kettenmann H. Acute insult of ammonia leads to calcium-dependent glutamate release from cultured astrocytes, an effect of pH. J. Biol. Chem. 280, 20937–44 (2005). 15802262 10.1074/jbc.M412448200

[b31] Thastrup O. . Thapsigargin, a novel molecular probe for studying intracellular calcium release and storage. Agents Actions 27, 17–23 (1989). 2787587 10.1007/BF02222186

[b32] Dibble C. C. & Cantley L. C. Regulation of mTORC1 by PI3K signaling. Trends Cell Biol. 25, 545–55 (2015). 26159692 10.1016/j.tcb.2015.06.002 PMC4734635

[b33] Vander Haar E., Lee S.-I., Bandhakavi S., Griffin T. J. & Kim D.-H. Insulin signalling to mTOR mediated by the Akt/PKB substrate PRAS40. Nat. Cell Biol. 9, 316–23 (2007). 17277771 10.1038/ncb1547

[b34] Ma X. M. & Blenis J. Molecular mechanisms of mTOR-mediated translational control. Nat. Rev. Mol. Cell Biol. 10, 307–18 (2009). 19339977 10.1038/nrm2672

[b35] Hirai H. . MK-2206, an allosteric Akt inhibitor, enhances antitumor efficacy by standard chemotherapeutic agents or molecular targeted drugs *in vitro* and *in vivo*. Mol. Cancer Ther. 9, 1956–67 (2010). 20571069 10.1158/1535-7163.MCT-09-1012

[b36] Berchtold D. . Plasma membrane stress induces relocalization of Slm proteins and activation of TORC2 to promote sphingolipid synthesis. Nat. Cell Biol. 14, 542–7 (2012). 22504275 10.1038/ncb2480

[b37] Kippenberger S. . Mechanical stretch stimulates protein kinase B/Akt phosphorylation in epidermal cells via angiotensin II type 1 receptor and epidermal growth factor receptor. J. Biol. Chem. 280, 3060–7 (2005). 15545271 10.1074/jbc.M409590200

[b38] Sedding D. G. . Caveolin-1 facilitates mechanosensitive protein kinase B (Akt) signaling *in vitro* and *in vivo*. Circ. Res. 96, 635–42 (2005). 15731459 10.1161/01.RES.0000160610.61306.0f

[b39] Diz-Muñoz A. . Membrane Tension Acts Through PLD2 and mTORC2 to Limit Actin Network Assembly During Neutrophil Migration. PLoS Biol. 14, e1002474 (2016). 27280401 10.1371/journal.pbio.1002474 PMC4900667

[b40] Nomura W. & Inoue Y. Methylglyoxal activates the target of rapamycin complex 2-protein kinase C signaling pathway in Saccharomyces cerevisiae. Mol. Cell. Biol. 35, 1269–80 (2015). 25624345 10.1128/MCB.01118-14 PMC4355542

[b41] Zinzalla V., Stracka D., Oppliger W. & Hall M. N. Activation of mTORC2 by association with the ribosome. Cell 144, 757–68 (2011). 21376236 10.1016/j.cell.2011.02.014

[b42] Patel P. R. . Identification of potent Yes1 kinase inhibitors using a library screening approach. Bioorg. Med. Chem. Lett. 23, 4398–403 (2013). 23787099 10.1016/j.bmcl.2013.05.072 PMC3769177

[b43] Tai Y.-L., Chen L.-C. & Shen T.-L. Emerging roles of focal adhesion kinase in cancer. Biomed Res. Int. 2015, 690690 (2015). 25918719 10.1155/2015/690690 PMC4396139

[b44] Hamamura K. . Functional activation of Src family kinase yes protein is essential for the enhanced malignant properties of human melanoma cells expressing ganglioside GD3. J. Biol. Chem. 286, 18526–37 (2011). 21454696 10.1074/jbc.M110.164798 PMC3099669

[b45] Lim S.-T., Mikolon D., Stupack D. G. & Schlaepfer D. D. FERM control of FAK function: implications for cancer therapy. Cell Cycle 7, 2306–14 (2008). 18677107 10.4161/cc.6367 PMC2574722

[b46] Wu N. . Marine bromophenol bis (2,3-dibromo-4,5-dihydroxy-phenyl)-methane inhibits the proliferation, migration, and invasion of hepatocellular carcinoma cells via modulating β1-integrin/FAK signaling. Mar. Drugs 13, 1010–25 (2015). 25689564 10.3390/md13021010 PMC4344615

[b47] Yang L. . Twist promotes reprogramming of glucose metabolism in breast cancer cells through PI3K/AKT and p53 signaling pathways. Oncotarget 6, 25755–69 (2015). 26342198 10.18632/oncotarget.4697 PMC4694864

[b48] Kappler M. . Normoxic accumulation of HIF1α is associated with glutaminolysis. Clin. Oral Investig, doi: 10.1007/s00784-016-1780-9 (2016). 26955835

[b49] Semenza G. L. HIF-1: upstream and downstream of cancer metabolism. Curr. Opin. Genet. Dev. 20, 51–6 (2010). 19942427 10.1016/j.gde.2009.10.009 PMC2822127

[b50] Powis K. & De Virgilio C. Conserved regulators of Rag GTPases orchestrate amino acid-dependent TORC1 signaling. Cell Discov. 2, 15049 (2016). 27462445 10.1038/celldisc.2015.49 PMC4860963

[b51] Hahn K. . PP2A regulatory subunit PP2A-B’ counteracts S6K phosphorylation. Cell Metab. 11, 438–44 (2010). 20444422 10.1016/j.cmet.2010.03.015

[b52] Liu J., Stevens P. D., Li X., Schmidt M. D. & Gao T. PHLPP-mediated dephosphorylation of S6K1 inhibits protein translation and cell growth. Mol. Cell. Biol. 31, 4917–27 (2011). 21986499 10.1128/MCB.05799-11 PMC3233022

[b53] Plescher M., Teleman A. A. & Demetriades C. TSC2 mediates hyperosmotic stress-induced inactivation of mTORC1. Sci. Rep. 5, 13828 (2015). 26345496 10.1038/srep13828 PMC4642562

[b54] Betz C. . Feature Article: mTOR complex 2-Akt signaling at mitochondria-associated endoplasmic reticulum membranes (MAM) regulates mitochondrial physiology. Proc. Natl. Acad. Sci. USA 110, 12526–34 (2013). 23852728 10.1073/pnas.1302455110 PMC3732980

[b55] Zhou X. . Dynamic Visualization of mTORC1 Activity in Living Cells. Cell Rep.doi: 10.1016/j.celrep.2015.02.031 (2015). PMC4567530 25772363

[b56] Carraro-Lacroix L. R., Jaumouillé V., Fairn G. D. & Grinstein S. A weak base-generating system suitable for selective manipulation of lysosomal pH. Traffic 12, 1490–500 (2011). 21819499 10.1111/j.1600-0854.2011.01266.x

[b57] Sun R. . Ammonium chloride inhibits autophagy of hepatocellular carcinoma cells through SMAD2 signaling. Tumour Biol. 36, 1173–7 (2015). 25342595 10.1007/s13277-014-2699-x

[b58] Tardito S. . Glutamine synthetase activity fuels nucleotide biosynthesis and supports growth of glutamine-restricted glioblastoma. Nat. Cell Biol. 17, 1556–68 (2015). 26595383 10.1038/ncb3272 PMC4663685

[b59] Krall A. S. & Christofk H. R. Rethinking glutamine addiction. Nat. Cell Biol. 17, 1515–7 (2015). 26612572 10.1038/ncb3278

[b60] Magasanik B. & Kaiser C. A. Nitrogen regulation in Saccharomyces cerevisiae. Gene 290, 1–18 (2002). 12062797 10.1016/s0378-1119(02)00558-9

[b61] Merhi A. & André B. Internal Amino Acids Promote Gap1 Permease Ubiquitylation via TORC1/Npr1/14-3-3-Dependent Control of the Bul Arrestin-Like Adaptors. Mol. Cell. Biol. 32, 4510–22 (2012). 22966204 10.1128/MCB.00463-12 PMC3486192

[b62] Boeckstaens M. . Identification of a Novel Regulatory Mechanism of Nutrient Transport Controlled by TORC1-Npr1-Amu1/Par32. PLoS Genet. 11, e1005382 (2015). 26172854 10.1371/journal.pgen.1005382 PMC4501750

[b63] Boeckstaens M., Llinares E., Van Vooren P. & Marini A. M. The TORC1 effector kinase Npr1 fine tunes the inherent activity of the Mep2 ammonium transport protein. Nat. Commun. 5, 3101 (2014). 24476960 10.1038/ncomms4101

[b64] Palkova Z. . Ammonia mediates communication between yeast colonies. Nature 390, 532–536 (1997). 9394006 10.1038/37398

[b65] Lorenz M. C. & Heitman J. The MEP2 ammonium permease regulates pseudohyphal differentiation in Saccharomyces cerevisiae. EMBO J 17, 1236–1247 (1998). 9482721 10.1093/emboj/17.5.1236 PMC1170472

[b66] Hess D. C., Lu W., Rabinowitz J. D. & Botstein D. Ammonium toxicity and potassium limitation in yeast. PLoS.Biol. 4, e351 (2006). 17048990 10.1371/journal.pbio.0040351 PMC1609136

[b67] Marini A. M. . The human Rhesus-associated RhAG protein and a kidney homologue promote ammonium transport in yeast. Nat.Genet. 26, 341–344 (2000). 11062476 10.1038/81656

[b68] Marini A. M., Urrestarazu A., Beauwens R. & Andre B. The Rh (rhesus) blood group polypeptides are related to NH4+ transporters. Trends Biochem. 22, 460–461 (1997). 10.1016/s0968-0004(97)01132-8 9433124

[b69] Biver S. . A role for Rhesus factor Rhcg in renal ammonium excretion and male fertility. Nature 456, 339–343 (2008). 19020613 10.1038/nature07518

[b70] Merhi A., De Mees C., Abdo R., Victoria Alberola J. & Marini A. M. Wnt/β-Catenin Signaling Regulates the Expression of the Ammonium Permease Gene RHBG in Human Cancer Cells. PLoS One 10, e0128683 (2015). 26029888 10.1371/journal.pone.0128683 PMC4452261

[b71] Goodwin M. L., Gladden L. B., Nijsten M. W. N. & Jones K. B. Lactate and cancer: revisiting the warburg effect in an era of lactate shuttling. Front. Nutr. 1, 27 (2014). 25988127 10.3389/fnut.2014.00027 PMC4428352

[b72] Parks S. K., Chiche J. & Pouysségur J. Disrupting proton dynamics and energy metabolism for cancer therapy. Nat. Rev. Cancer 13, 611–23 (2013). 23969692 10.1038/nrc3579

