# Supplementary material for: The metabolic waste ammonium regulates mTORC2 and mTORC1 signaling
Source: Sci Rep. 2017 Mar 17;7:44602. doi: 10.1038/srep44602 (PMC5355986; doi:10.1038/srep44602)

Supplementary Information

The metabolic waste ammonium regulates mTORC2 and mTORC1 signaling

Ahmad Merhi[1,2,3#], Paul Delrée[2,3], Anna Maria Marini[1#]

1. Biology of Membrane Transport, IBMM, Université Libre de Bruxelles, Rue des Professeurs Jeener et Brachet 12, 6041 Gosselies, Belgium

2. Institute of Pathology and Genetics, Avenue Georges Lemaître 25, 6041 Gosselies, Belgium.

3. Tumour Bank, Institute of Pathology and Genetics, Avenue Georges Lemaître 25, 6041 Gosselies, Belgium.

Supplementary Figure S1. Uncropped images of the immunoblots presented in the main figures.

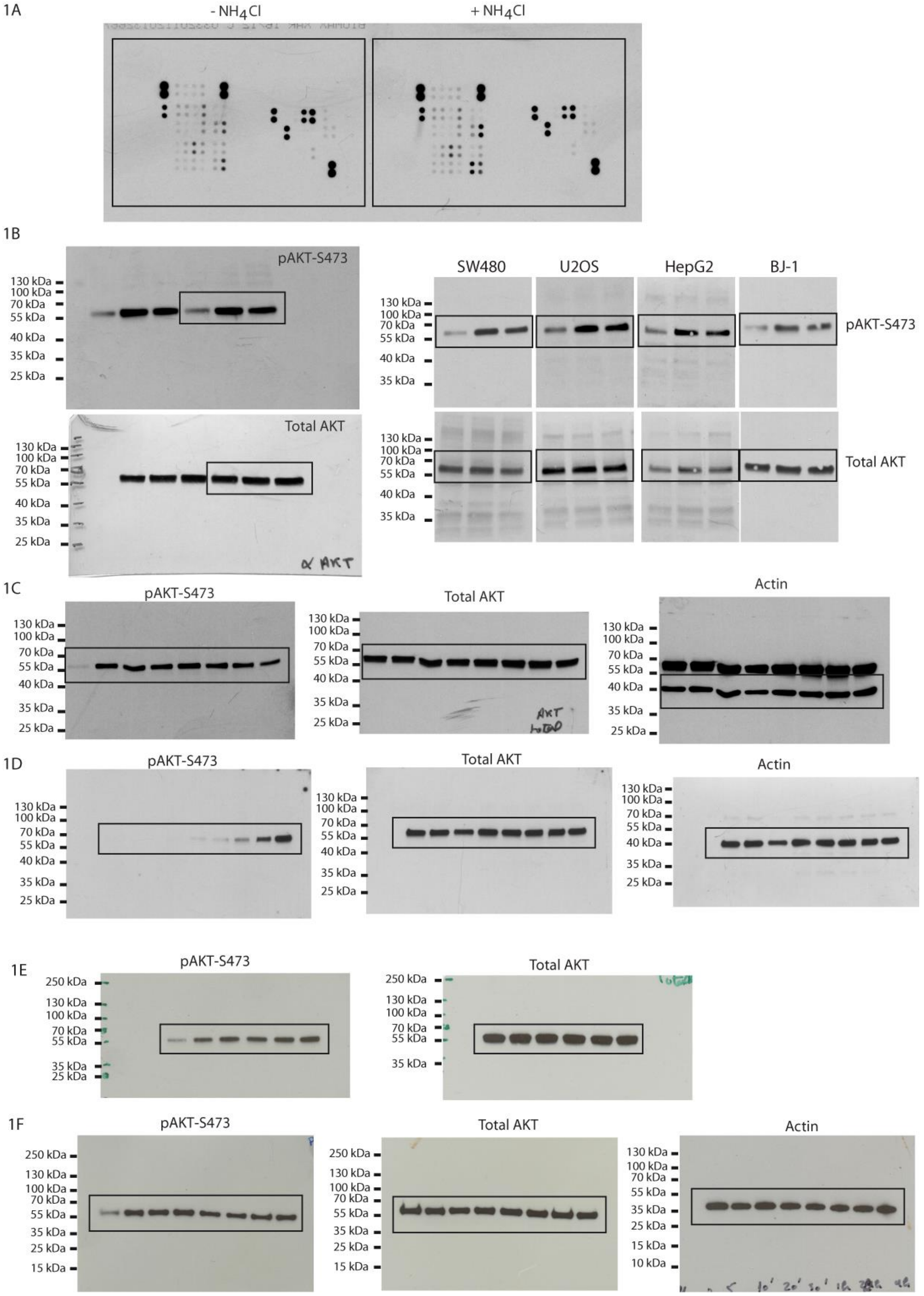

(Continued)

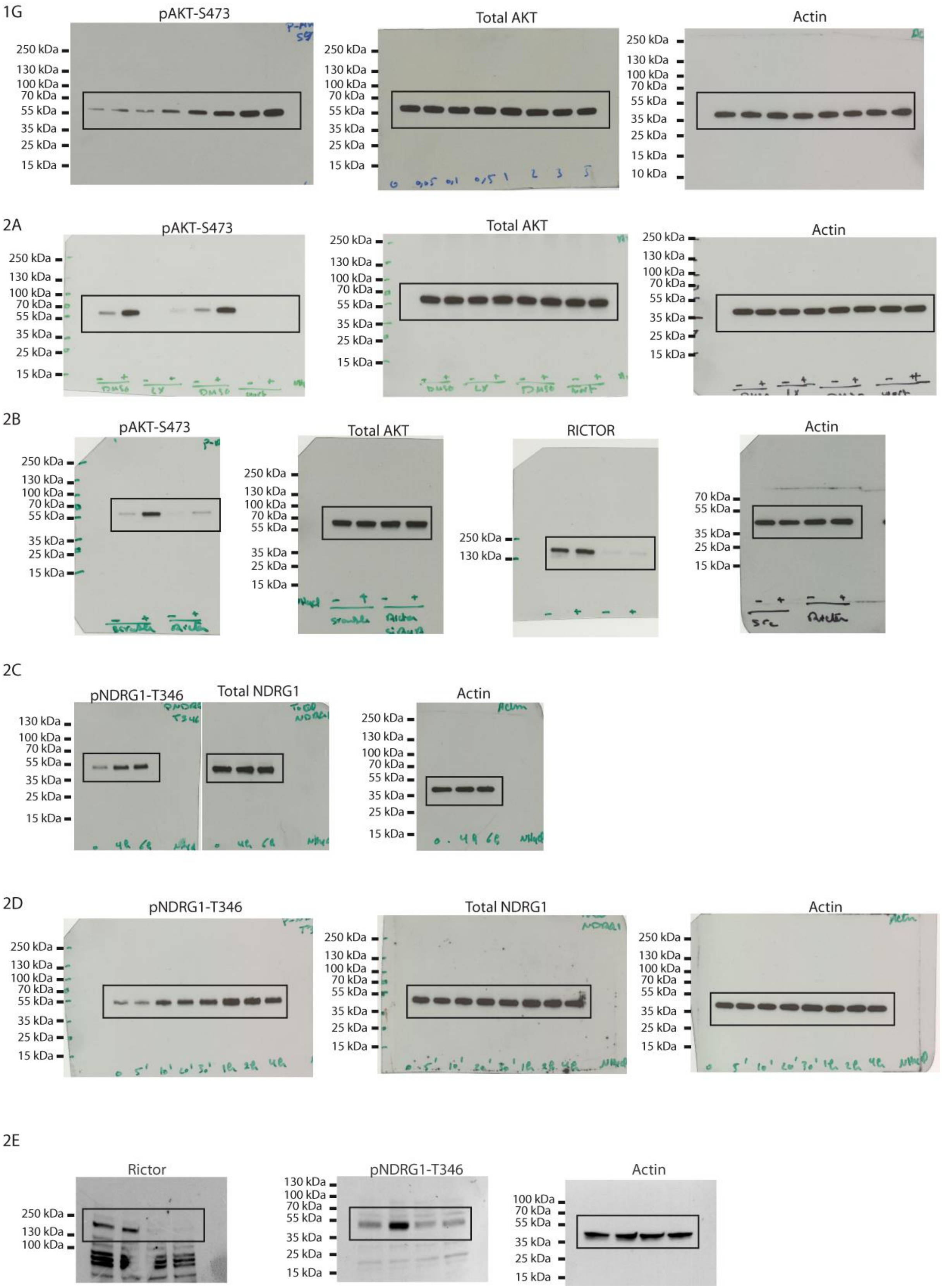
1G
pAKT-S473
Total AKT
Actin
250 kDa
130 kDa
100 kDa
70 kDa
55 kDa
35 kDa
25 kDa
15 kDa
10 kDa
2A
pAKT-S473
Total AKT
Actin
2B
pAKT-S473
Total AKT
RICTOR
Actin
2C
pNDRG1-T346
Total NDRG1
Actin
2D
pNDRG1-T346
Total NDRG1
Actin
2E
Rictor
pNDRG1-T346
Actin

(Continued)

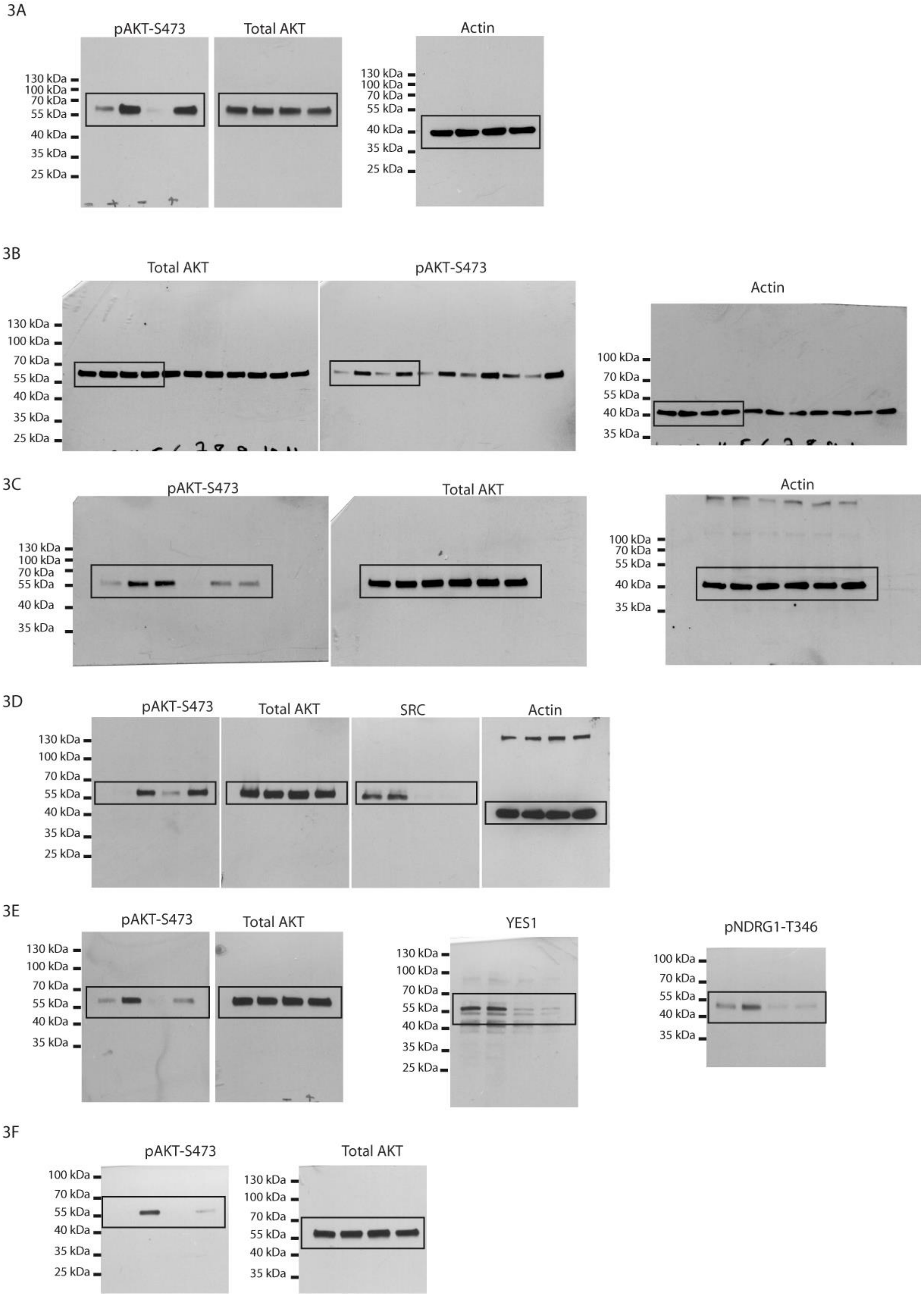
3A
pAKT-S473
Total AKT
Actin
3B
Total AKT
pAKT-S473
Actin
3C
pAKT-S473
Total AKT
Actin
3D
pAKT-S473
Total AKT
SRC
Actin
3E
pAKT-S473
Total AKT
YES1
pNDRG1-T346
3F
pAKT-S473
Total AKT
130 kDa
100 kDa
70 kDa
55 kDa
40 kDa
35 kDa
25 kDa

(Continued)

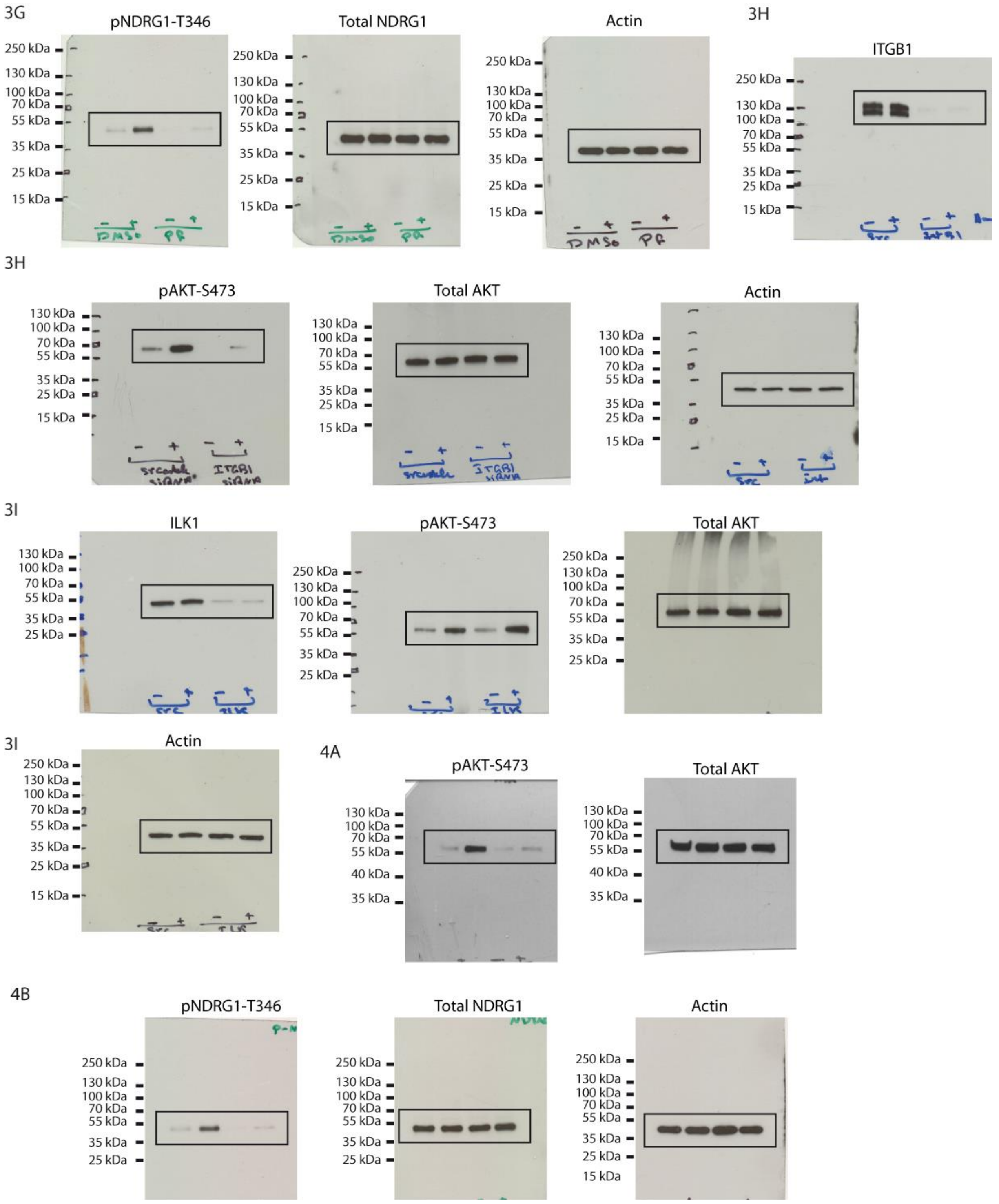
3G
pNDRG1-T346
Total NDRG1
Actin
3H
ITGB1
3H
pAKT-S473
Total AKT
Actin
3I
ILK1
pAKT-S473
Total AKT
3I
Actin
4A
pAKT-S473
Total AKT
4B
pNDRG1-T346
Total NDRG1
Actin
250 kDa
130 kDa
100 kDa
70 kDa
55 kDa
40 kDa
35 kDa
25 kDa
15 kDa

(Continued)

4C

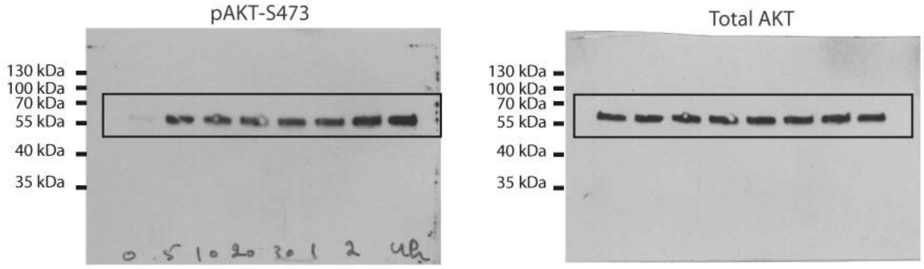

4D

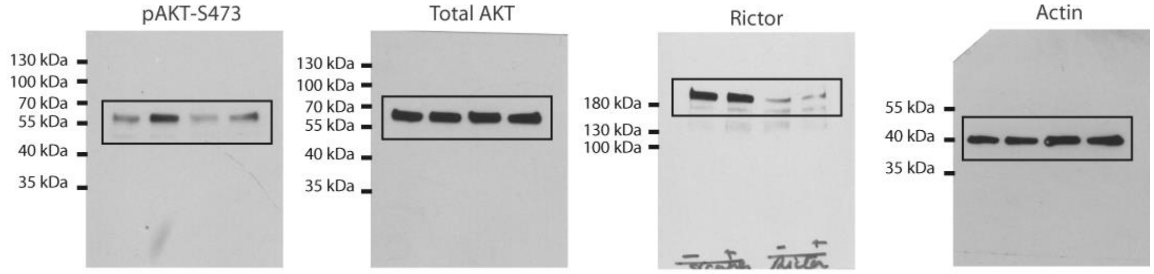

5A

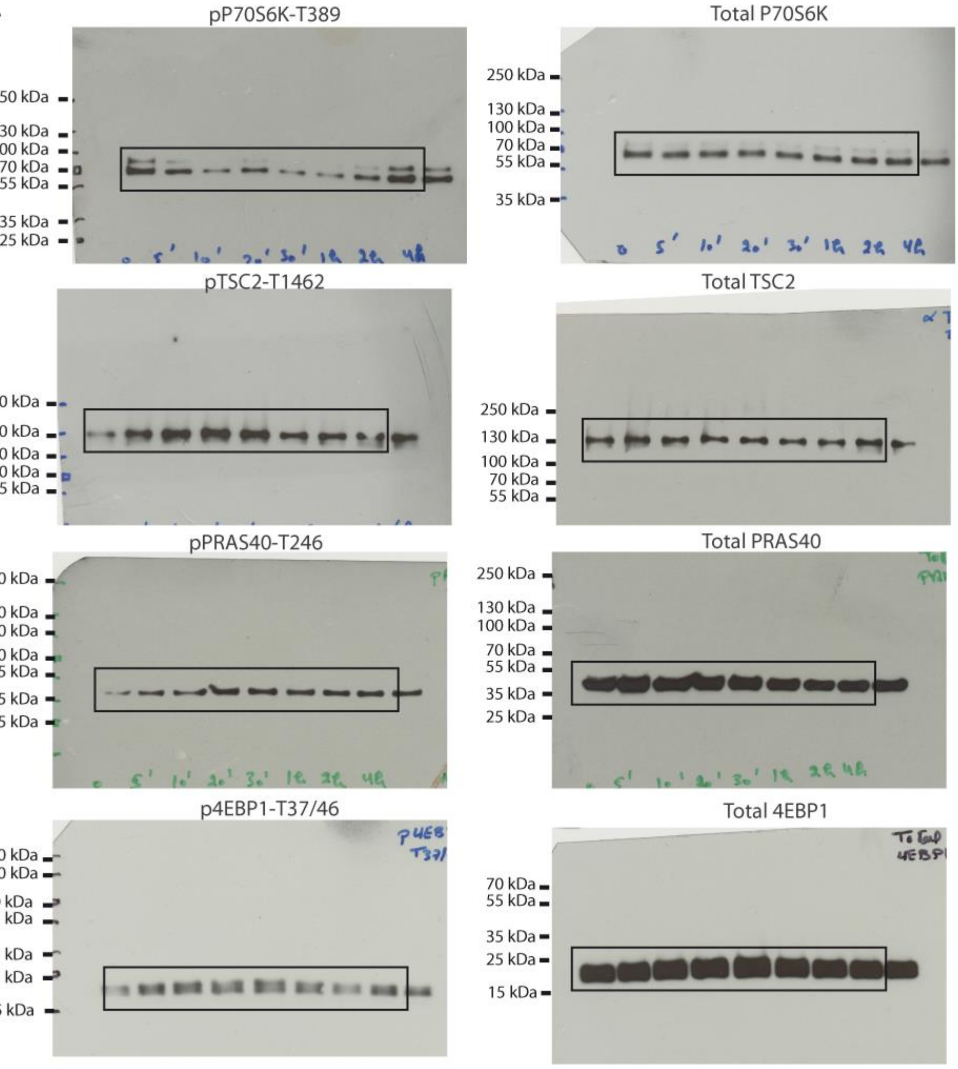

(Continued)

5A

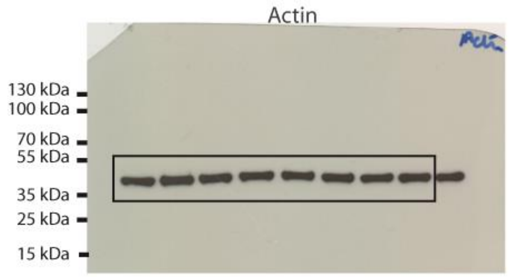

5B

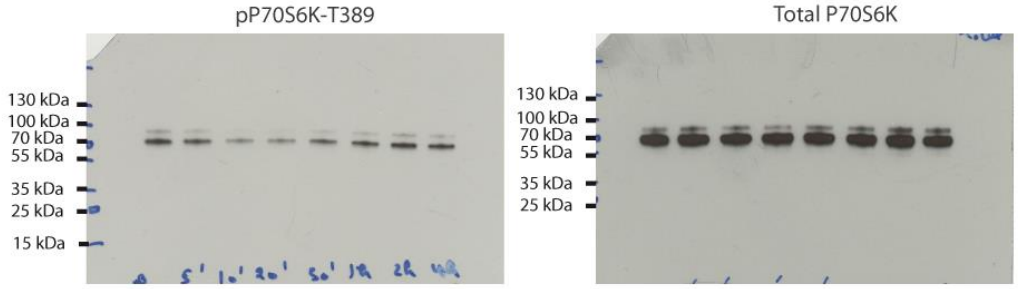

5C

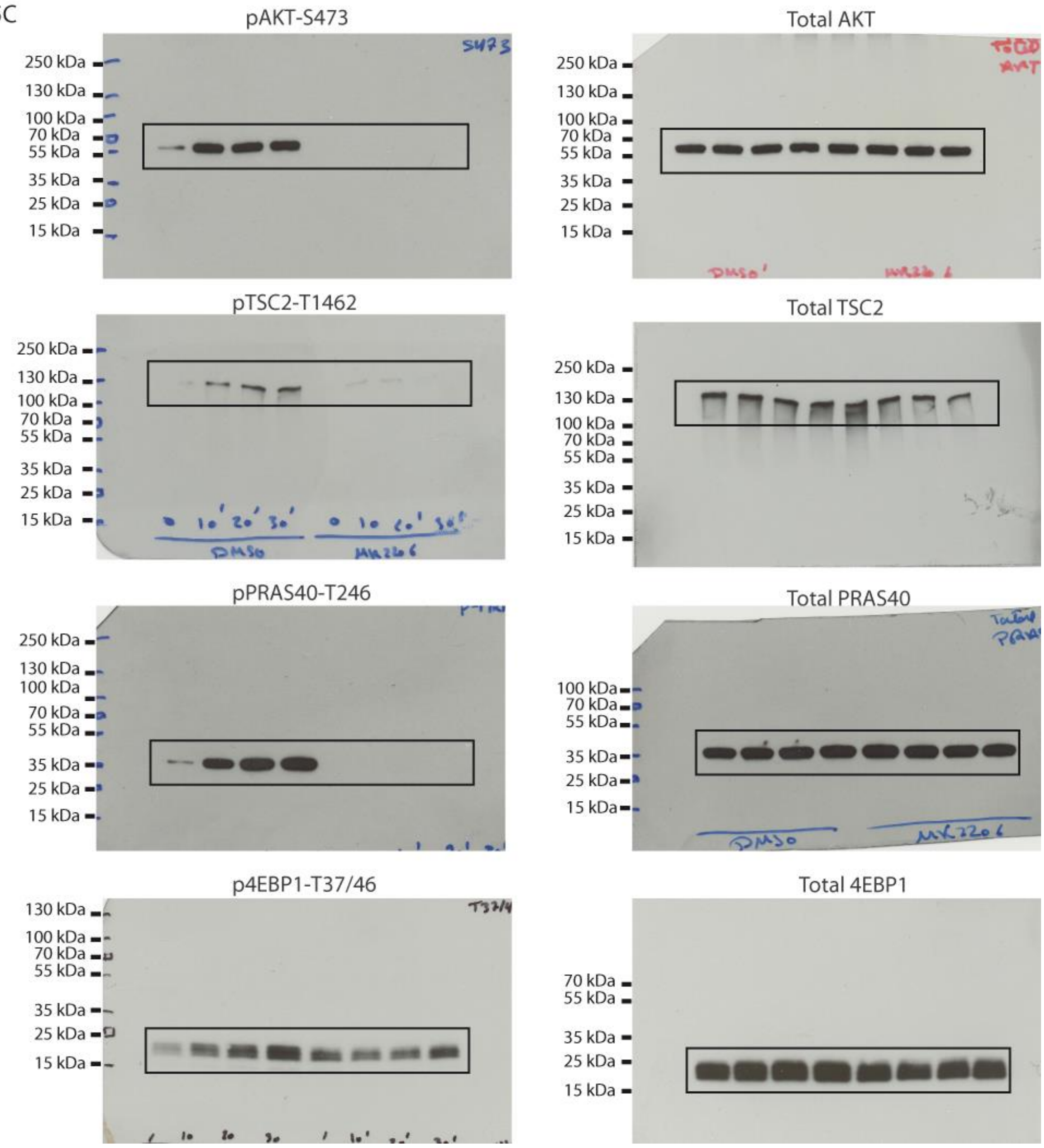

(Continued)

5C

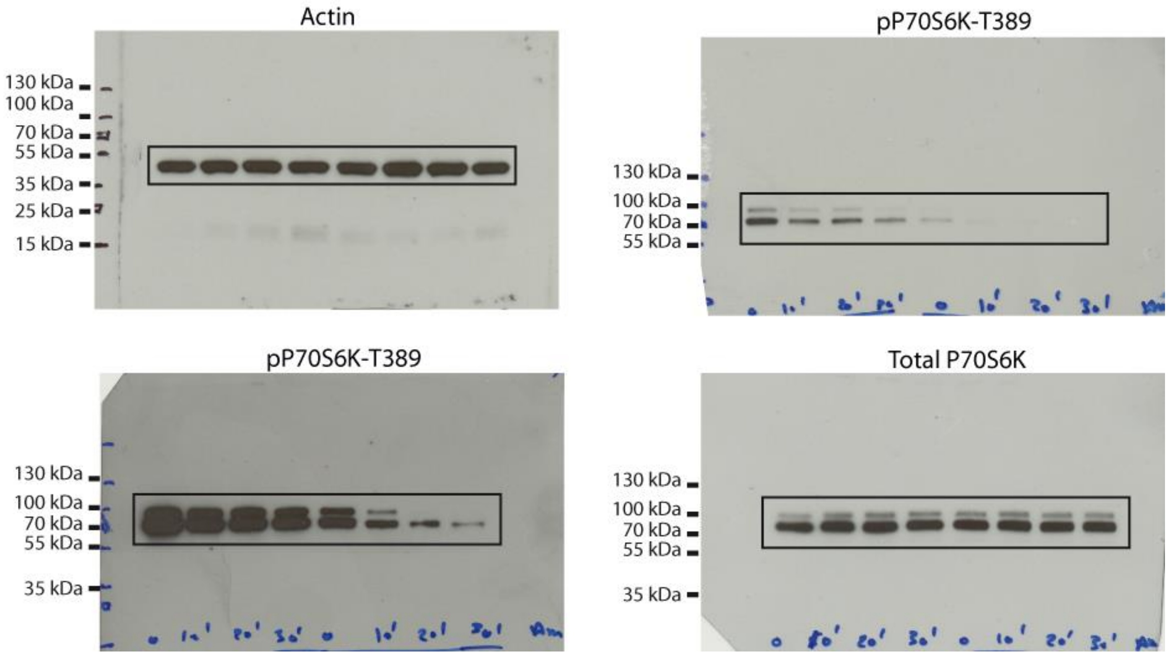

Supplement: Supplementary Information [file srep44602-s1.pdf]
